# Trans-Scale Insights into Variability in Radiation Cancer Risk Across Tissues, Individuals, and Species

**DOI:** 10.3390/biology14081025

**Published:** 2025-08-09

**Authors:** Tatsuhiko Imaoka

**Affiliations:** 1 Department of Radiation Effects Research, Institute for Radiological Science, National Institutes for Quantum Science and Technology (QST), Chiba 263-8555, Japan; imaoka.tatsuhiko@qst.go.jp; 2 Institute for Quantum Life Science, National Institutes for Quantum Science and Technology (QST), Chiba 263-8555, Japan; 3 Tokyo Metropolitan University, Tokyo 116-8551, Japan

**Keywords:** radiation effect, diversity, carcinogenesis, radiological protection, animal model, human

## Abstract

Diversity is evident in various aspects of life and the human population. The current radiological protection system considers the diversity in disease susceptibility and incorporates some of it to provide a unified risk model for the human population. This paper focuses on variability in cancer risk among cells/tissues, individuals, and animal species. At the cell/tissue level, radiation cancer risk per unit dose varies among tissues, as indicated by epidemiological and animal studies. This variation is most likely due to the epigenetics of cells. At the individual level, radiation risks vary considerably depending on physiological, environmental, and genetic factors. At the species level, epidemiology is considered the most relevant source of radiological information, and limitations have been recognized in extrapolating animal data to human risks. A recent study demonstrated a fundamental relationship between cancer risk and somatic mutation rate in humans and mice. This relationship could lead to a new extrapolation method, which could be used to protect a wider range of species, including humans and other mammals. Thus, considering diversity in radiation cancer risk provides insight into the underlying aspects of radiobiology of radiological protection.

## Simple Summary

Ionizing radiation affects the health of humans and animals. One of its harmful effects is an increased risk of cancer. The same amount of ionizing radiation increases the risk of cancer differently in different organs, people, and animal species (including humans). This review paper summarizes examples of this diversity based on knowledge from studies of humans and experimental animals. To effectively use radiation while protecting people from its harmful effects, it is important to consider the different responses of human organs, as well as the differences among people of different ages, sexes, lifestyles, living environments, and genetics. The current radiological protection considers this diversity and incorporates some of it within its system, but the consideration may need to be continuously reevaluated based on the latest knowledge. Understanding diversity between animal species will contribute to the application of the results of animal experiments on radiation cancer risks to the protection of humans, pets, livestock, and wildlife animals.

## 1. Introduction

Radiation is the emission of physical energy from nuclear reactions, radionuclides, the action of radiation on materials, and man-made devices. It is emitted in the form of photons and other energetic particles. Ionizing radiation refers to radiation with the capacity to ionize atoms. All life is exposed to natural radiation, which is typically present at very low levels. Space is abundant in radiation and radionuclides continuously produced by stellar activity. However, the Earth’s geomagnetic field and atmosphere protect it from radiation, and the long time period between the formation of terrestrial radionuclides and the emergence of life has kept the current level of natural radiation very low. The discovery of radiation is a relatively recent event in human history, dating back only to 1895. This discovery was a significant event in the history of science and technology, leading to the use of nuclear energy and the development of radiological protection. Indeed, radiation is harmful to life. Just as clothing was necessary for our ancestors to enter cold regions, radiological protection is indispensable for us to use radiation and explore space. Thanks to radiological protection, human and environmental exposure to radiation is generally low. Acute radiation injury and various radiation-induced tissue reactions have a threshold dose below which no effects occur. Of concern in low-dose and low-dose-rate exposures are cancer and hereditary effects, which are referred to as stochastic effects. A low dose is typically defined as a dose below 100 mGy, whereas a low dose rate generally refers to a dose rate below 0.1 mGy/min averaged over about an hour [1,2]. Thus, radiobiology forms one of the foundations of radiological protection. 

Diversity is evident in many aspects of life. Biology attempts to record, analyze, and integrate this diversity to construct a unified view. Every organism is composed of diverse molecules, and multicellular organisms have various cell types and organs that result in diverse biological functions. The number of species in an ecosystem is another aspect of diversity. Diversity also refers to natural differences within the human population. The scope of this present paper is to describe examples of diversity related to radiation cancer risk in a biological context, explain their mechanistic origins, and discuss the significance of considering the diversity at different levels. The paper focuses on cancer as the most relevant low dose effect in humans and some laboratory animals.

## 2. Mechanism and Risk of Radiation Carcinogenesis

### 2.1. Early Radiation Actions, DNA Damage, and Repair

**Figure 1:**
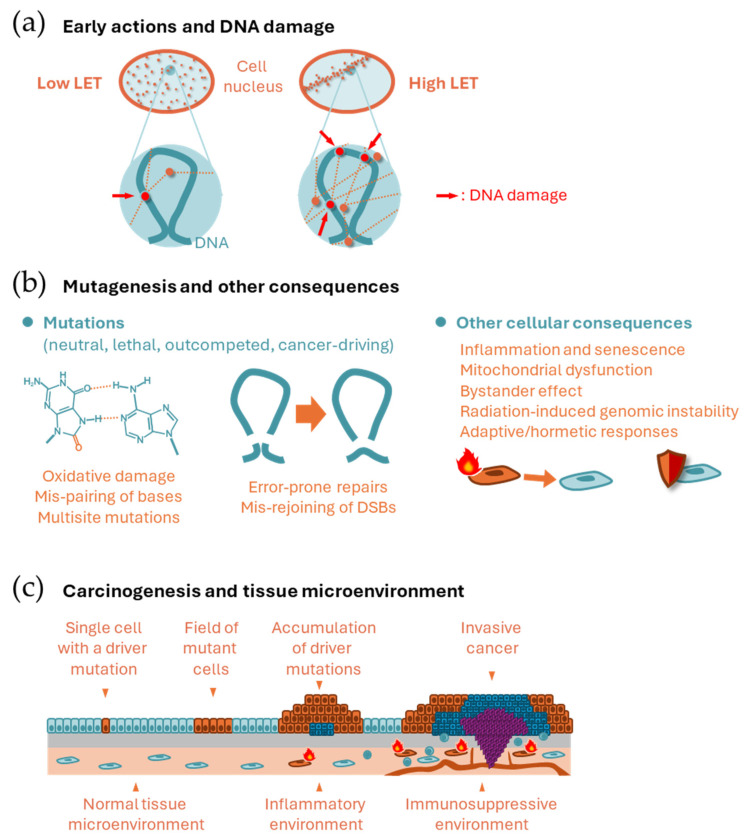
Scheme of mechanisms of radiation carcinogenesis. (**a**) Spatial distribution of energy deposition (dots) along radiation tracks (doted lines) by low and high LET radiation at the cellular scale (**upper**) and nanoscale (**lower**). (**b**) Mutagenesis and other cellular consequences. (**Left**) Mispairing of 8-oxoguanine and adenine, which leads to CG → TA transversion. Aggregation of such mutations in nearby bases leads to multisite mutations. (**Center**) Mis-rejoining of DNA ends resulting from multiple DSBs leading to structural variations. (**Right**) Radiation-related cellular consequences other than mutagenesis. (**c**) Epithelial (**upper**) and microenvironmental (**lower**) changes during carcinogenesis.

At the nanoscale, radiation deposits energy only along its track, rather than uniformly, resulting in a heterogeneous distribution of ionization (Figure 1a). This ionization generates scattered electrons that directly affect DNA and other biomolecules or ionize water molecules to produce free radicals. Free radicals are highly reactive and initiate chain redox reactions in nearby molecules, including water and biomolecules. Therefore, radiation can damage DNA and other biomolecules directly and indirectly (via free radicals).

Linear energy transfer (LET) refers to the density of energy deposited by radiation along its path. Examples of low-LET radiation include photons (γ rays and X rays), electrons, and protons, whereas particle radiation, such as α particles, heavy ions, and neutrons, are examples of high-LET radiation. The LET of radiation depends on its energy, and those radiation types classified as low LET above sometimes are considered as having higher LET (e.g., Auger electrons and low-energy protons). At the microscopic level relevant to cellular size, the energy deposition of high LET radiation is heterogenous, whereas the energy deposition of low LET radiation is more uniform (Figure 1a). An identical physical dose (in Gy) thus has a stronger biological effect when the LET is high. Relative biological effectiveness (RBE) is defined as the ratio of the physical dose of reference radiation (usually photons) to the dose of a radiation type of interest that produces an identical biological effect. To account for differences in biological effects, the radiation dose is often weighted by the RBE (RBE-weighted dose) or by coefficients that reflect differences in RBE (e.g., radiation quality factor and radiation-weighting factor).

DNA is exceptionally important because it carries unique information necessary for cellular functions. Therefore, DNA damage can sometimes be critical for a cell. This contrasts with other types of biomolecules, such as RNA, proteins, and lipids. DNA is double-stranded, which serves as a backup for the information it carries. In most cases of single-strand damage, the cellular DNA repair system can restore the information using the backup. However, if both strands are damaged simultaneously, the correct information could be lost forever. Thus, radiobiology often focuses on DNA double-strand breaks (DSBs). Several repair mechanisms exist for DSBs, including canonical non-homologous end joining, homologous recombination, alternative end joining, and single-strand annealing [3]. Nonhomologous end joining can be error-prone unless the broken DNA ends have a defined chemical structure. Other systems, except homologous recombination, are also error-prone. Simulation studies have predicted that locally dense energy deposition from radiation can produce complex (or clustered) DNA damage, which is a more difficult type of damage to repair [4].

### 2.2. Mutagenesis and Other Cellular Consequences

Mutations can be induced by radiation and other spontaneous or external processes, such as thermal instability, replication errors, natural oxidative stress, and endogenous/exogenous mutagenic substances. Some of these mutations have specific nucleotide-sequence contexts related to their origins. The contexts are referred to as mutational signatures. For example, cancers associated with smoking and ultraviolet radiation exhibit distinct mutational signatures [5]. Radiation-induced oxidation of the DNA molecule often manifests as oxidized guanine residues due to the high reductive potential of guanine; mispairing involving these damaged residues leads to specific types of single nucleotide variations [6] (Figure 1b, left). In addition, incorrect rejoining of DSBs often leads to deletion mutations of a few to millions of bases, chromosomal inversions, and translocations (Figure 1b, center). Large-scale changes of this type are collectively referred to as structural variations. Probably due to clustered DNA damage, multisite mutations (i.e., small, adjacent mutations within several bases) are often reported as radiation-induced changes [7,8]. While most mutations may be neutral, some may disadvantage or benefit the survival and proliferation of the mutant cell and its progeny. If the affected cells survive, they are detected as mutant cells. Mutations that contribute to cellular transformation are called cancer driver mutations, while neutral mutations found in cancer are called passenger mutations. Driver mutations typically affect protooncogenes and tumor suppressor genes. Conversely, cells with a certain repertoire of mutations have lowered fitness within a cell population and are outcompeted by neighboring healthy cells. This process is called cell competition and provides a protective mechanism against cancer [9].

Cells with oncogenic mutations sometimes acquire senescence-like phenotypes, including arrested proliferation and the release of inflammatory signals that prompt the immune system to attack them [10]. Irradiated cells may also produce signals in the form of extracellular vesicles [11]. DNA DSBs and their repair can cause epigenetic changes and these changes can induce a senescence-like state [12]. If a DNA fragment persists until mitosis and is not properly redistributed in the daughter cell nuclei, it can form a micronucleus. This type of cytosolic DNA can be sensed by the cellular defense mechanism against pathogens, inducing an inflammatory response [13]. In addition, oxidative stress and other signals may induce fragmentation and dysfunction of mitochondria, leading to further production of oxidative stress and nuclear DNA damage [14]. The ‘bystander’ effect of radiation refers to changes induced in cells that are not irradiated by themselves. The relevance of senescence-associated phenotypes and inflammation in bystander effects is not established. Radiation is known to induce a type of genetic instability (radiation-induced genomic instability), which may involve unknown mechanisms including epigenetic changes and mitochondrial dysfunction [15,16].

Some evidence suggests that low levels of radiation can have adaptive and beneficial effects on cells (Figure 1b, right). Many cell lines exhibit high radiosensitivity at doses of several tens of mGy and increased resistance above a few hundred mGy, reaching saturation of resistance above ~1 Gy [17]. The phenomenon in which prior radiation exposure of cells to radiation at levels of a few to several tens of mGy reduces defects caused by exposure to 1 Gy or more hours later is known as the radiation adaptive response [18]. A hypothesis for this mechanism is that radiation induces efficient DNA repair above a certain dose within a timescale equivalent to DNA damage removal. Some evidence supports the beneficial effects of low dose radiation without subsequent high-dose exposure, which is collectively called radiation hormesis [19].

### 2.3. Carcinogenesis and the Role of the Tissue Microenvironment

Normal cells reproduce daily by cell division and cooperate with surrounding cells to maintain tissue function and organ size. Cancer cells proliferate autonomously and leave their original niche to invade surrounding tissues. Most cancers originate from a single cell [20] (Figure 1c). Carcinogenesis is a multistage phenomenon in which normal cells become cancerous by acquiring multiple driver mutations [21]. Pathologically, cancer arises through multiple stages of precancerous lesions, which represent clones of cells that accumulate different numbers of driver mutations [21] (Figure 1c). Driver mutations are also observed in normal-appearing human tissues [22], demonstrating the expansion of clones harboring driver mutations before they are pathologically identifiable. It is unclear what “single cell” is the origin of the cancer. Many normal tissues have long-lived stem cells that give rise to differentiated cells via progenitor cells. It may be these tissue stem/progenitor cells that acquire multiple genetic alterations to give rise to cancer, although differentiated cells may acquire stemness through genetic alterations in some cases [23].

The tissue microenvironment also plays crucial roles in carcinogenesis (Figure 1c). Chronic inflammation promotes carcinogenesis, as evidenced by the high cancer risk associated with ulcerative colitis (colon cancer), hepatitis (liver cancer), *Helicobacter pylori* infection (stomach cancer), and asbestos-related bronchitis (lung cancer) [23]. Thus, cancer can be prevented by suppressing chronic inflammation, and in fact, regular use of an anti-inflammatory drug is associated with a decreased risk of colorectal cancer [23]. The immune system may also promote carcinogenesis, for example, through immunosuppressive tissue microenvironments such as those with elevated numbers of regulatory T cells [23]. Cellular senescence is thought to be a mechanism that inhibits cancer development. However, it has also been suggested that the number of senescent cells increases with age and that a state called senescence-associated secretory phenotype, in which cells secrete inflammatory proteins, may create a tissue microenvironment that promotes carcinogenesis [24]. Although radiation can induce various microenvironmental changes (as mentioned above), there is insufficient evidence to determine the role of these changes in radiation carcinogenesis.

### 2.4. Mathematical Models of Carcinogenesis and Risk Expression

**Figure 2:**
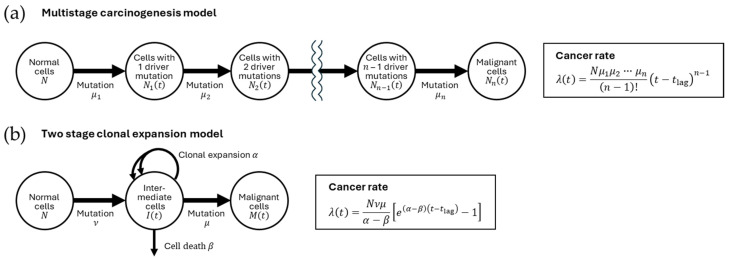
Mathematical model of carcinogenesis. (**a**) Multistage carcinogenesis model [25]. Normal cells (population size, *N*) generate cells with one driver mutation (population size, *N*_1_(*t*) at time *t*) at a rate of *μ*_1_. These cells in turn generate cells with two driver mutations (population size, *N*_2_(*t*)) at a rate of *μ*_2_. *n* repetitions of such processes generate malignant cells (population size, *N_n_*(*t*)). (**b**) Two-stage clonal expansion model [26]. Normal cells generate intermediate cells at a rate of *ν*. Intermediate cells (population size, *I*(*t*)) have a higher net clonal expansion rate (i.e., the difference between expansion rate *α* and the death rate *β*) than normal cells. The intermediate cells generate malignant cells (population size, *M*(*t*)) at a rate *μ*. In both (**a**) and (**b**), mutation rates are assumed to be so low that the reduction in the number of cells prior to mutation is negligible, and cancer diagnosis is assumed to follow the appearance of a malignant cell after a lag time *t*_lag_. The cancer rate *λ*(*t*) (i.e., the probability of having cancer in a unit time) is formulated as in the boxes.

Figure 2 shows representative schemes of the carcinogenesis process that provide a basis for mathematical modeling. In a multistage carcinogenesis model [25], the accumulation of driver mutations is considered as rate-limiting; under this assumption, the cancer rate (i.e., the probability of having cancer in a unit time) is proportional to the cell population size, mutation rates, and a power of the age (Figure 2a). The two-stage clonal expansion model is also often used to describe carcinogenesis [26]. This model assumes an advantage of premalignant cells over normal cells in clonal expansion, which serves as a rate-limiting step, and further assumes two important steps (i.e., acquisition of clonal expansion ability and acquisition of malignancy); the cancer rate is proportional to the cell population size, the mutation rates for the two steps, and an exponential function of age (Figure 2b). Although various extensions and combinations are possible, these two are the most fundamental and provide insight into the role of factors (i.e., cell population size, mutation, and clonal expansion) that may modify the cancer rate. Radiation exposure may alter one or more of the mutation rates, the clonal expansion rate, or both, and its effect may be only during exposure, for some time after exposure, or permanent.

**Figure 3:**
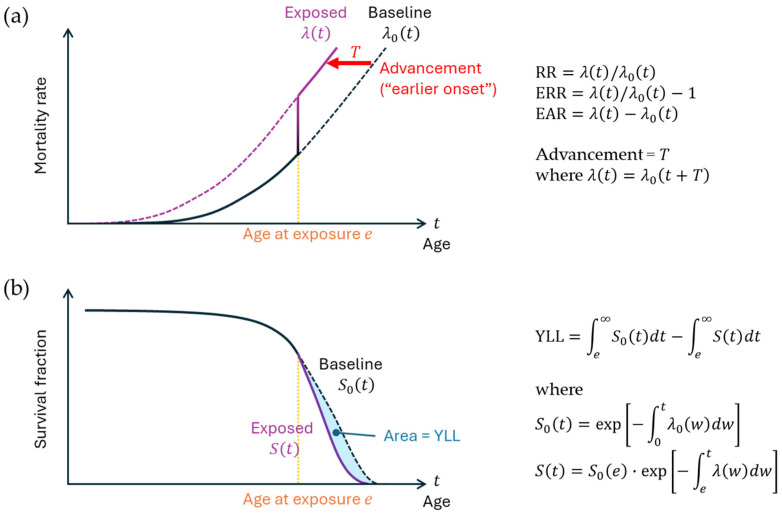
Risk measures in epidemiology. (**a**) Mortality rate and the definitions of relative risk (RR), excess relative risk (ERR), and excess absolute risk (EAR). The mortality rate denotes the probability of a living person’s death at a given time. *λ*_0_(*t*) and *λ*(*t*) represent the idealized mortality rate functions for the nonexposed and exposed groups, respectively, at attained age *t*, where exposure occurs at age *e*. The vertical line indicates the transition from the nonexposed state to the exposed state. Advancement (*T*) signifies the extent to which the function shifts to the left due to exposure. (**b**) Survival curve and the definition of years of life lost (YLL). *S*_0_(*t*) and *S*(*t*) represent survival functions for the nonexposed and exposed groups, respectively; these functions are defined by the mortality rates, as shown in the equations on the right. The lag time between exposure and the manifestation of its effect is omitted. The overall concept on mortality herein can be applied to disease incidence.

In epidemiology, there are several common ways to express exposure-related cancer risk (Figure 3). One commonly used measure is relative risk, which is the ratio of the cancer rate in the exposed group, λ(t), to the rate in the unexposed population, *λ*_0_(*t*), i.e., *λ*(*t*)/*λ*_0_(*t*). Excess relative risk (ERR) refers to the exposure-related component of relative risk, or *λ*(*t*)/*λ*_0_(*t*) − 1. Excess absolute risk (EAR) refers to the net increase in the cancer rate (*λ*(*t*) − *λ*_0_(*t*)). Note that EAR = ERR × *λ*_0_(*t*). A recent discussion suggests that the effect of radiation is understood as advancement of the manifestation of cancer risk in time [27] (*T* in Figure 3a). The advancement of solid cancer has been estimated to be 5 years per Gy in Japanese atomic bomb survivors (sex-averaged) exposed at age 30 [28]. Years of life lost refers to the reduction in life expectancy due to exposure (Figure 3b). Previously, an estimation of 1.38 years of life lost per Gy from solid cancer has been made [29].

## 3. Diversity at the Cell/Tissue Scale

The first discussion is about the diversity of radiation-related cancer risk among tissues, which are composed of different types of cells.

### 3.1. Diversity in the Magnitude of Radiation Cancer Risk Among Tissues

**Figure 4:**
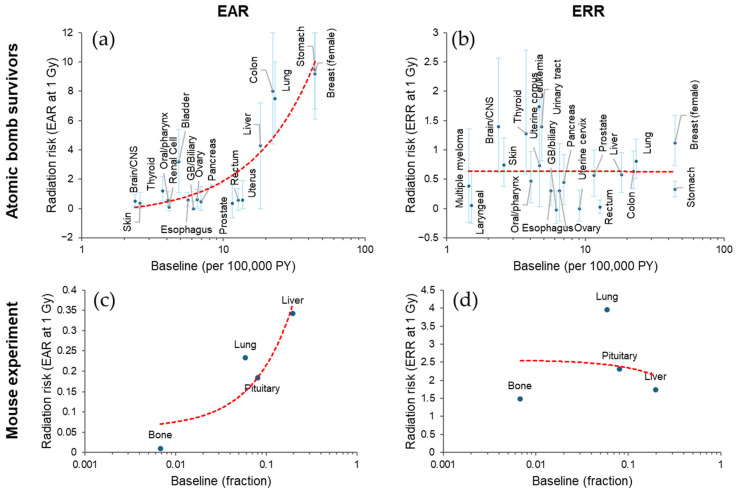
Diversity of radiation-related cancer risk among tissues. (**a**,**b**) Excess absolute risk (EAR, per 100,000 person-years [PY]) with 90% confidence interval [30] and excess relative risk (ERR) with 95% confidence interval [31,32,33,34,35,36,37,38,39,40,41,42,43], respectively, for cancer incidence in Japanese atomic bomb survivors at attained age 70 after exposure at 1 Gy at age 30 (except for thyroid, for which the risk is at attained age 60 after exposure at age 10). Baseline incidence is based on another source [44], from which 1995 data were used (except for breast cancer, for which 2005 data were used due to lack of 1995 data). (**c**,**d**) Tumor mortality EAR and ERR, respectively, at 1 Gy in female B6C3F1 mice [46], where EAR was calculated by multiplying baseline (per lifetime) and reported ERR. Red dashed lines in (**a**–**d**), linear fit. CNS, central nervous system. GB, gallbladder. See Supplementary File for the data used.

The studies of Japanese atomic bomb survivors indicate variation in both EAR and ERR per unit dose among tissues: high EAR is associated with cancers of the colon, lung, stomach, and female breast, whereas high ERR is associated with leukemia and cancers of the brain, thyroid, and urinary tract [30,31,32,33,34,35,36,37,38,39,40,41,42,43]. These trends are at least partially related to the baseline incidence rate (i.e., the incidence rate in the unexposed population). Plotted against the baseline incidence rate in Japan [44,45], a high EAR is associated with a high baseline incidence (Figure 4a). Conversely, some cancers with low baseline rates have high ERRs with large confidence intervals (Figure 4b); this is understandable given that the ERR is based on a relative risk (RR), which can be a ratio of a low rate in the exposed group to an even lower baseline rate. When the large confidence intervals are considered, the ERR per unit dose appears to be more consistent than the EAR between tissues, although some tissue differences may remain. If the ERR is more consistent, the fact that the EAR is related to the baseline is reasonable because the EAR is a product of the ERR and the baseline rate (see above).

The same trend is supported by an animal study [46]. In this study, the percentage of mice dying from a specific cause at the end of the observation period was fitted to a function of dose and adjusted for mortality from competing causes. The linear term of the dose response function was used as a proxy for ERR, and the product of ERR and baseline values was used as a proxy for EAR. The result shows an apparent positive association between EAR (but not ERR) and baseline (Figure 4c,d).

### 3.2. Diversity in Relative Biological Effectiveness (RBE) Among Tissues

Although it is common practice in radiological protection to represent the differential effect of high-LET radiation by a single weighting factor (see above), RBE often depends on the biological endpoint. For example, the RBE for skin reactions is generally larger than that for skin cell survival [47]. The biological context also influences the RBE for carcinogenesis. Animal experiments on the RBE of neutrons and carbon ions have shown different RBE values for different tissues (Table 1). Estimation of RBE by an epidemiological approach is generally challenging due to the low dose and resulting small risk magnitude [48,49], although efforts are being made on the Japanese atomic bomb survivor cohort [50,51] and patients receiving carbon ion radiotherapy [52].

### 3.3. Mechanisms of Diversity in Baseline Cancer Risk

#### 3.3.1. Tissue Stem Cell Activity

Regarding the baseline cancer rate, some cancers are quite common while others are rare. This inter-organ variation in cancer risk has been addressed by a series of mathematical analyses [65,66]. The study estimated the “total number of stem cell divisions” in various tissues and indicated that these numbers correlated well with the lifetime cancer risk of those tissues [65]. This correlation was consistent across countries, suggesting that the effect of environmental factors (which are supposed to differ between countries) is not substantial [66]. An experimental approach has supported the idea that the reproductive capacity of stem cells determines tissue cancer risk [67]. Herein, cells expressing the stem cell marker prominin and their progeny were fluorescence-labeled using a technique called cell lineage tracing. The prominin-marked clones showed varying degrees of expansion among tissue, and there was a good correlation between stem cell capacity and tumor incidence among organs of different genetically modified mice [67].

#### 3.3.2. Repertoire of Cancer Driver Genes/Mutations

The tissue-specific repertoire of cancer driver genes and mutations may be another source of diversity. A bioinformatic analysis has constructed a compendium of cancer driver genes across cancer types, revealing the heterogeneity in the size of the repertoire (e.g., 99 driver genes may play a role in breast cancer, 42 in lung adenocarcinoma, and 20 in neuroblastoma) [68]. Overall, 10 genes are capable of driving cancer in more than 20 tissues, while 360 genes act as drivers in only one or two tumor types [68]. Even the same driver genes play different roles in different types of cancer. For example, *EGFR* mutations in glioblastoma are mostly in the extracellular domain and stabilize the open conformation of the receptor to stimulate its autophosphorylation, whereas *EGFR* mutations in lung adenocarcinoma are often in the tyrosine kinase domain and act to increase the enzymatic activity [68]. The combinations of driver genes are heterogeneous among cancer types [69]. A subsequent analysis added that the order in which driver mutations accumulate is tissue-dependent and thus appears to be another source of variation [70].

In terms of the mathematical model, the cancer driver repertoire may be related to the mutation rates (*ν*, *μ*, *μ*_1_, …, and *μ_n_*), as some cancer driver genes have functions in DNA repair and genome maintenance. It may also be related to the clonal expansion rate *α*, which is the relative advantage of intermediate mutant cells over normal cells, as some cancer drivers fuel cell proliferation (see Figure 2b).

#### 3.3.3. Exposure

Apart from driver genes, the total number of mutations (including passenger mutations) in some cancers, such as skin, lung, bladder, liver, and the digestive tract cancers, is large, at least in part reflecting their high likelihood of external exposure [68]. This may point to environmental exposure as another source of organ variability in baseline cancer risk, although the aforementioned mathematical study has estimated that environmental exposure may account for at most one-third of the variation [66].

In the mathematical scheme (see Figure 2), continuous exposure to genotoxic agents may be associated with increased mutation rates (*ν*, *μ*, *μ*_1_, …, and *μ_n_*). Some chemicals may promote tumorigenesis by stimulating cell proliferation, which can be interpreted as increasing proliferation-related mutations and/or increasing the chance of clonal expansion for intermediate cells that have acquired growth advantage.

### 3.4. Mechanisms of Diversity in Radiation Effects

The roles played by radiation in carcinogenesis can be the induction of driver mutation(s) in cells that have survived radiation-induced killing and the induction of microenvironments that lead either to an increased mutation rate or to the advantage of cells with specific driver mutations in clonal expansion.

#### 3.4.1. Radiation-Induced Mutagenesis

Regarding mutation induction, similar frequencies of radiation-induced somatic mutations have been reported among cell types in cell culture studies [71]. Mutations are rare and usually result in reduced cellular fitness through loss of gene function, making radiation-induced mutations difficult to detect experimentally. An old and effective solution is to use genes that determine drug sensitivity, where loss of function of these genes results in cell survival in the presence of a drug, allowing analysis of mutant clones. In this setting, human, mouse, and hamster cells show similar levels of mutant yield [71]. More recently, single-cell sequencing technology has enabled exome and genome-wide analysis. A landmark study showed that irradiation of mammalian cells at 1 Gy results in ~2.33 mutations per Gb (including 2.15 insertion/deletions, 0.17 SVs, and 0.01 complex rearrangements) using mouse and human breast, colon, and pancreatic cells irradiated in vivo, despite a high degree of intercellular stochasticity [72]. Thus, the available evidence supports that radiation-induced mutation does not vary considerably between tissues. On the other hand, the ratio of the radiogenic mutation rate to the spontaneous rate (the latter possibly related to tissue differences in stem cell activity, cancer driver repertoire, and exposure) may be heterogeneous among tissues and determine the magnitude of the ERR per Gy.

#### 3.4.2. Radiation-Induced Microenvironmental Changes

In addition to mutation induction, there may be intercellular variability in DNA damage response, DNA repair mechanisms, radiosensitivity (i.e., survival) of stem cells, and possibly clonal expansion effects of radiation, resulting in a tissue microenvironment that varies between tissues. For instance, adult intestinal stem/progenitor cells respond to radiation by transient cell cycle arrest and induction of apoptosis, whereas adult liver cells typically do not proliferate and thus do not appear to respond [73,74]. Radiation-induced cell death may lead to compensation of cell loss by proliferation of surrounding cells in a tissue, and such compensation is likely to be competitive, i.e., cells with an advantageous driver mutation may preferentially undergo clonal expansion, a process called super-competition [75].

#### 3.4.3. Epigenetic Diversity

Such a cell type-specific response is likely to be determined by epigenetics, since all tissues in an individual should have an identical genome. In fact, even in the same tissue, different cells (e.g., basal and luminal cells of the mammary gland) exhibit different radiation response, apoptosis, and reproductive cell death [76,77], and epigenetic reprogramming alters the cellular radiation response [78].

In medical physics, RBE of high-LET radiation is understood as a result of the interaction between the microdosimetric distribution of radiation energy deposition and the physical substructure of the genome [79]. Such a biophysical model has recently been used to understand the RBE of different particle radiations in cellular transformation and induction of the Harderian gland tumor in mice [80]. Advances in genome biology have revealed the picture of three-dimensional (3D) physical substructures of the genome, which is closely related to the epigenetic mechanism of gene expression regulation. Therein, chromosomes are separated as chromosomal territories, and chromosomal regions are further separated as topologically associating domains defined by cohesin and CCCTC-binding factor, as well as loops defined by enhancer-promoter interactions [81]. Theoretically, looping of DNA by contact of two DNA regions should form a complex fractal-like structure [82]. Genomic regions of different chromosomes also interact in both universal and cell type-specific ways to determine cellular functions [83]. It is therefore expected that mis-rejoining of DNA ends generated by multiple DNA double strand breaks will result in losses and inversions, with DNA loops of different sizes as units, and translocations between different chromosomes. Since the 3D chromatin architecture depends on the cell type and the repertoire of cancer drivers varies between tissues, the probability of generating a driver mutation by radiation should depend on the cell/tissue type. The efficiency of high-LET radiation in inducing such consequences may also depend on the 3D chromatin structure (Figure 1a) and thus on the cell/tissue type, providing an explanation for the variation in RBE among tissues.

## 4. Diversity at the Individual Scale

Individual differences in radiation risk exist due to a myriad of physiological, environmental, and genetic factors. This poses a practical problem in estimating cancer risk from radiation exposure, since the choice between ERR and EAR models produces different risk estimates for populations of individuals with different baseline cancer risks (note that EAR = ERR × baseline). A comprehensive listing of modifying factors is not the focus here, as such work is ongoing [84,85,86]. This section briefly mentions examples of modifying factors and discusses possible underlying biological mechanisms.

### 4.1. Physiological Modifiers of Radiation Cancer Risk

#### 4.1.1. Sex

**Figure 5:**
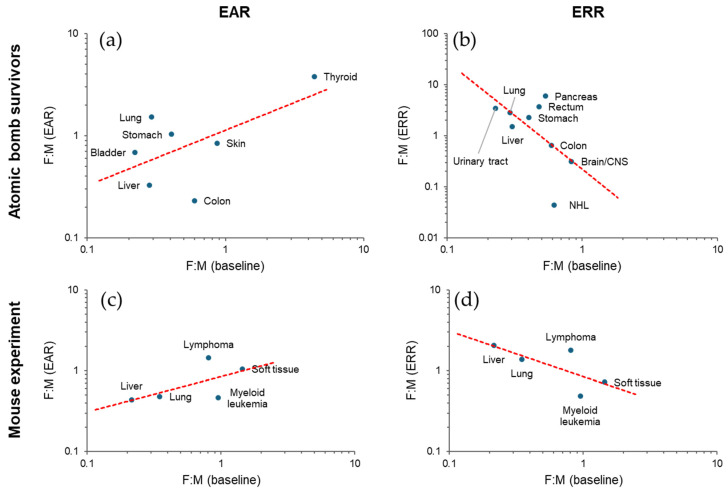
Sex ratio relationship between baseline and radiation risk. (**a**,**b**) Female-to-male (F:M) ratio in excess absolute risk (EAR) [30] and excess relative risk (ERR) [31,32,33,34,35,36,37,38,39,40,41,42,43], respectively, for cancer incidence in Japanese atomic bomb survivors at attained age 70 after exposure at 1 Gy at age 30 (except for thyroid, which is at attained age 60 after exposure at age 10) plotted against baseline incidence F:M of the Japanese population in 1995 [44]. (**c**,**d**) F:M ratios of tumor mortality analyzed by EAR- and ERR-based approaches, respectively, in a cohort of HS/Npt mice [88]. ERR-based here refers to Cox’s approach, whereas EAR was calculated by multiplying the reported sex ratios of the baseline and radiation-related components. Dashed lines, power function fit. See Supplementary File for the data used.

Cancer risk is inherently sex-dependent. In the non-smoking, non-radiation-exposed population of the Japanese atomic bomb survivor cohort, men are more susceptible to solid cancer than women at cancer age [87]. Fitting to linear dose response models indicated that the radiation-related risk is significantly higher in women in terms of ERR (female-to-male ratio 1.81, 95% confidence interval [CI] 1.42 to 2.35), and only marginally higher in terms of EAR, for age 30 at exposure and attained age 70 [87]. The ERR/Gy for breast cancer is 1.12 (95% CI 0.73 to 1.59) for women and 5.7 (95% CI 0.3 to 30.8) for men at age 70 exposed at age 30 [36], whereas the baseline incidence is more than 100 times higher in women. In general, the EAR and ERR sex ratios correlate positively and negatively, respectively, with the baseline sex ratio between tissues (Figure 5a,b). An analysis of an animal experiment [88] shows a similar trend (Figure 5c,d).

#### 4.1.2. Age at the Time of Exposure

Increasing age at exposure generally decreases the risk of radiation-related cancer. The epidemiology of Japanese atomic bomb survivors shows a decreasing trend in ERR/Gy (−22% per decade) and EAR/Gy (−30% per decade) with increasing age at exposure. An experiment in female B6C3F1 mice also indicates a decreasing trend in ERR/Gy (coefficient −2.6/year in a log-linear function, corresponding to −19% per month) [89]. Although not necessarily statistically significant, a decreasing trend with increasing age at exposure has been suggested for the risk of rectal [32], liver [33], brain and central nervous system [41], and thyroid [42] cancers, and an increasing trend for colon cancer [32], lung cancer [34], and myeloid leukemia [43]; a peak around menarche has been suggested for breast [36] and uterine corpus [37] cancers. These trends are mostly supported by animal studies (decreasing trends for liver [90], brain [54], and thyroid [91] cancers; increasing trend for lung cancer [92] and myeloid leukemia [64]; a peripubertal peak for breast cancer [93]).

#### 4.1.3. Reproductive Status/History

Early age at menarche increases the ERR/Gy for breast cancer in Japanese atomic bomb survivors [36]. A history of childbirth decreases breast cancer in rats irradiated before puberty [94], and treatments with estrogens increase [95,96], while those with an anti-estrogen and estrogens with weak activity decrease [97], radiation-related breast cancer in rats.

#### 4.1.4. Chronic Inflammation

Chronic disease status is one of the most important considerations in the diversity of individuals. Evidence suggests that chronic inflammation may contribute to the modulation of radiation cancer risk. An investigation on the Adult Health Study cohort of Japanese atomic bomb survivors has suggested a supra-multiplicative interaction between radiation exposure and hepatitis virus C infection regarding the risk of liver cancer [98]. Animal studies have shown that induced inflammation enhances radiation-induced mandibular gland tumors [99], gastric cancer [100], myeloid leukemia [101], and colon cancer [102] in mice.

### 4.2. Environmental Modifiers of Radiation Cancer Risk

#### 4.2.1. Physical Environment

In addition to ionizing radiation itself, the physical environment relevant to cancer risk includes non-ionizing radiation, temperature, and gravity. Overall, there is insufficient evidence about how these factors modify radiation cancer risk.

*Ultraviolet.* Ultraviolet, a non-ionizing radiation, is a risk factor for non-melanoma skin cancer. An epidemiological study showed a higher EAR/Gy in the sun-exposed scalp margin than in the relatively sun-protected scalp [103] and a lower ERR/Gy for the face and neck (more likely to be exposed to sunlight) than for the rest of the body [35]. An animal study suggests a combinatorial effect of X rays and simulated solar radiation in inducing skin cancer in hairless mice [104].

*Temperature.* Temperature is related to cancer in that the risk of cancer is high in countries with cold temperatures [105,106] and that frequent consumption of hot beverages can be a risk factor for upper aerodigestive tract cancer [107]. Animal studies have shown that cold environmental temperature suppresses the growth of tumor xenografts [108] and that hyperthermia enhances radiation carcinogenesis [109,110,111].

*Gravity.* Spaceflight imposes several stresses including microgravity and space radiation. It is unclear whether microgravity alone induces cancer in humans. Microgravity induces bone density loss, muscle atrophy, and fluid shifts toward the upper body, which may affect various organ systems [112]. An animal experiment has shown that simulated microgravity enhances radiation-induced intestinal tumorigenesis in a manner that deviates from both additivity and multiplicativity, probably by impairing the immune system [113].

#### 4.2.2. Chemical Environment

The environment contains various chemical substances that may increase the risk of cancer. There is evidence that some chemicals modify radiation cancer risk.

*Smoking.* Tobacco smoking modulates the radiation dose response of lung cancer risk in Japanese atomic bomb survivors in a complex manner [34]. The effect of passive smoking on radiation cancer risk has not been studied directly, but the higher radiation-related lung cancer risk in female atomic bomb survivors may be due to a multiplicative interaction between passive smoking and radiation [114]. Cigarette smoke exposure interacts with plutonium dioxide exposure in a supra-additive manner to induce lung cancer in rats [115].

*Food-borne chemicals.* Heterocyclic amines are a class of chemicals formed in cooked meat and fish [116], and 1-methyl-1-nitrosourea is formed from fish source and nitrite in the stomach [117]. These substances induce mammary cancer in rats and interact with γ rays in a multiplicative manner [93]. 1-Ethyl-1-nitrosurea, a model chemical used to induce mutations in laboratory organisms, shows a supra-additive interaction with X rays in the induction of T-cell lymphoma in mice [118]. Note that these examples are genotoxic chemicals that interact with DNA and induce mutations.

*Tumor promoter compounds.* Some chemicals act as tumor promoters by stimulating tissue cell proliferation, providing a microenvironment that favors cell clones with cancer driver mutation(s). Carbon tetrachloride and chloroform act as promoters of liver tumor in mice, and carbon tetrachloride increases, but chloroform does not increase, liver tumors in neutron-irradiated mice [119]. 12-*O*-tetradecanoylphorbol-13-acetate and β radiation act additively as promoters of skin tumors in mice [120].

#### 4.2.3. Biological Environment

*Diet/nutrition.* Consumption of green-yellow vegetables and fruits reduces radiation-related cancer mortality in Japanese atomic bomb survivors in a manner that did not deviate from additivity or multiplicativity [121]. Caloric restriction in laboratory mice reduces radiation-related increases in myeloid leukemia [122], liver cancer [123], and intestinal tumors [124]. Some dietary components are protective against tumors in experimental animals exposed to radiation. For example, curcumin reduces mammary tumors in irradiated rats [125]; dietary antioxidants and soybean protease inhibitor reduce the development of lymphoma and other neoplastic lesions in irradiated mice [126]. Some vitamins and minerals may have the opposite effect. Experimental studies have shown an increased risk of cancer in irradiated mice treated with vitamin A [127] and those with iodine deficiency and overdose [128].

*Infection and microbiota.* In Japan, human T-cell leukemia virus is more prevalent in some regions including Nagasaki, but not in Hiroshima. The Japanese atomic bomb survivor study has indicated a higher EAR for leukemia in Nagasaki (with Hiroshima-to-Nagasaki ratio of 0.52, 95% CI 0.26 to 0.93), suggesting a modulation of radiation-related leukemia risk [43]. As mentioned above, hepatitis C virus infection modifies the risk of liver cancer in Japanese atomic bomb survivors [98]. Activation of a leukemogenic virus by radiation has been reported in some strains of mice [129]. In an old experimental study, radiation-induced myeloid leukemia in mice was reduced when they were kept in a germ-free environment [130]. More recently, the gut microbiome of mice was modulated by administering a cocktail of antibiotics for 6 weeks before and after radiation exposure, and these mice were found to be more susceptible to radiation-induced solid cancer mortality than a radiation-only control, which was not the case when antibiotics were administered 1 month after exposure [131]. Thus, infections and microbiota may modulate radiation-related cancer risk.

### 4.3. Genetic Modifiers of Radiation Cancer Risk

#### 4.3.1. Ancestry and Genetic Polymorphism

Cancer risk differs among people of different ancestry due to inherent genetic polymorphisms as well as variations in lifestyle, socioeconomic status, and access to health care [132]. Many reported single nucleotide polymorphisms and copy number variations are associated with ancestral diversity, although their biological and clinical significance is largely unknown [132]. In general, the vast majority of these genetic polymorphisms may confer only minor predispositions, while a small number of them have large effects on disease susceptibility. Some studies have identified associations between genetic polymorphism and radiation cancer risk. In cancer survivors who received radiotherapy for Hodgkin lymphoma, the risk of second cancer is associated with single nucleotide polymorphisms at rs4946728 and rs1040411 related to the *PRDM1* gene [133]. Breast cancer risk after radiotherapy for childhood cancer of the chest is associated with single nucleotide polymorphisms in rs4342822, rs74949440, and rs17020562, which are located near *PROX1*, *TAGLN*, and *RPS6KC1*, respectively [134].

#### 4.3.2. Animal Strain

A strain of a laboratory animal refers to a group that shares a specific genetic makeup and is distinguishable from other groups, and thus, animal strains are sometimes compared to human ancestry. Strains are often developed through selective breeding and/or genetic modification, and some strains are more susceptible to certain cancers than other strains, whether they are spontaneous or induced [135]. For example, the C57BL/6 strain of mice is more susceptible to radiation-induced thymic lymphoma than the CBA strain, and vice versa for radiation-induced myeloid leukemia [136]; the Copenhagen rat is more resistant to radiation-induced mammary cancer than the Sprague-Dawley rat [137]. A study of radiation carcinogenesis in mice of systematically mixed strains showed a significant effect of familial relatedness on radiation cancer risk [87], revealing the effect of strain-related genetic makeup in a highly genetically diverse population.

#### 4.3.3. Specific Genetic Variations

As mentioned above, only a small number of genetic variations in the human population have a major impact on disease susceptibility. Studies have provided some evidence for an association between radiotherapy-associated second cancer risk and the deleterious variants of *RB1* [138], *ATM* [139], *TP53* [140], and *BRCA2* [141], but the evidence is generally not strong. Animal studies have suggested significant effects of genetic alterations in *Trp53* [142] and *Nf1* [143] on tumors overall, *Ptch1* on medulloblastoma [144], *Apc* on intestinal [145] and mammary [146] tumors, and *Brca1* on breast [147] and ovarian tumors [148] induced by radiation. Animal studies have failed to demonstrate the effect of *Atm* mutations on radiation-induced tumors [149,150].

### 4.4. Mechanisms of Diversity

The aforementioned diversity among individuals may be mechanistically determined by individual differences in target cell number, initial cell death induced by radiation exposure, long-term clonal expansion, and baseline mutation rate, as discussed above for diversity among cells/tissues.

#### 4.4.1. Physiological Diversity

The cell number can be influenced by sex in sex-associated organs and by parity in the mammary gland [151], and thus underlies the sex- and parity-associated modification of baseline and radiation cancer risk. The somatic mutation rate may also depend on sex. There is a sex-related bias in somatic mutations in cancer [152], although the underlying mechanism is unclear. Proposed mechanisms include sex differences in DNA repair mechanisms such as those for DNA double strand breaks [153].

Regarding the modification by age at exposure, the developing tissues of perinatal rodents contain specific undifferentiated/proliferative cells that are the origins of radiation-induced medulloblastoma and renal cell cancer [154,155], where the cell number is the relevant mechanism. Intestinal cells of young mice and bone marrow cells of adult mice are refractory to radiation-induced cell death, which explains their susceptibility to radiation-induced intestinal tumors and myeloid leukemia at these ages [73,156]. Alternatively, the expansion of tissue stem cells in young organisms (e.g., crypt fission in the intestine [157] and terminal end bud branching in the mammary gland [158]) may provide an opportunity for clonal expansion of cells with a driver mutation. Radiation induces cell proliferation in some tissues (e.g., the thymus [159] and the liver [74]), a process that can depend on age.

#### 4.4.2. Environmental Diversity

The suppression of radiation leukemogenesis by caloric restriction may be mediated in part by the reduced number of hematopoietic progenitor cells [160]. Otherwise, there is little evidence to support the relevance of cell number changes in the environmental modification of radiation cancer risk. Theoretically, two carcinogens acting at the same step in carcinogenesis alter cancer risk in an additive manner, two carcinogens acting at different steps alter risk in a more synergistic manner; in particular, two agents acting on first mutation and clonal expansion, respectively, may interact in a supra-multiplicative manner [161,162,163]. In fact, many physical, chemical, and biological agents can affect the mutational and clonal expansion phases of carcinogenesis, and radiation can act in both ways, as detailed above. The interaction of radiation with caloric restriction on liver carcinogenesis is additive [123] and interpreted as two agents acting on lipid-induced inflammation as a tumor-promoting microenvironment [164]. The interaction between radiation and genotoxic chemicals on thymic lymphomagenesis and mammary carcinogenesis is supra-additive to multiplicative [93,118], and there is evidence for radiation-induced clonal expansion of chemically initiated cells [165,166]. The supra-multiplicative interaction of radiation and high corn oil diet on mammary carcinogenesis could be interpreted as their respective effects on initial mutation via DNA double strand breaks and clonal expansion via prostaglandin-related inflammation [93,167].

#### 4.4.3. Genetic Diversity

Ancestry is known to influence the prevalence of cancer driver mutations, including the *EGFR* mutation in lung cancer (more prevalent in Asians) and *PTEN* deletion in prostate cancer (more prevalent in Caucasians) [132]. Driver mutations in radiogenic cancers remain understudied [168], and it is not known whether this diversity underlies the ancestral effect on radiation cancer risk. More has been learned about the variation in radiation cancer risk among animal strains. Using a series of congenic mouse strains between BALB/c and STS strains, a study has compared DNA DSB repair capacity and radiation-induced lung cancer risk and found a strong correlation [169]. Thus, DNA DSB repair capacity is one factor that accounts for the differential radiation cancer risk. However, more complex biology may be involved, as the strain susceptibility to radiation-induced cancer varies among tissues [170]. Distinct susceptibility loci have been identified for radiation-induced lymphoid and myeloid neoplasms [171,172]. While these loci may represent relatively low-penetrance mutations in tumor suppressor genes, various gene knockout animals serve as models for extremely high-penetrance mutations leading to high radiation cancer risk [142,143,144,145,146,147].

## 5. Diversity at the Animal Species Scale

### 5.1. Cancer in Non-Human Animals

Diversity in radiation cancer risk among animal species is related to at least three issues: extrapolation from animals to humans, animal welfare, and environmental protection. First, animal studies play an essential role in the assessment of human cancer risks from radiation, although species differences hinder the direct application of experimental results to the estimation of human risks, raising the issue of animal-to-human extrapolation. Second, there has been an increase in the use of radiation in the health care of pets and livestock, necessitating radiological protection in the veterinary field [173]. With the commercialization of space travel and the expansion of human society into the extraterrestrial environment, pets and livestock may be taken into space and exposed to space radiation, providing another opportunity for animal radiological protection. Third, although cancer has not generally been considered as a conservation concern, it may be relevant in at least some species [174,175]. Animals in human care develop tumors primarily before their natural lifespan, and in some animals such as reptiles, cancer is not related to age [176]. In an effort to consider the effects of tritium beta rays and alpha particles on non-human biota, the International Commission on Radiological Protection reviewed experiments on various species, examining endpoints such as early mortality, reduced reproductive success, morbidity (tumors, organ atrophy and cell death), chromosomal damage and mutation [177].

### 5.2. Diversity in Baseline Cancer Risk Among Animal Species

As explained above, mathematical models (see Figure 2) predict that a larger body size (i.e., a greater number of somatic stem cells) will lead to a higher incidence of cancer. In reality, however, although whales weigh 1000 times more than humans, their cancer risk is not as high, and mice weigh 1000 times less, their cancer risk is not as low. Animals seem to have a comparative level of susceptibility to cancer despite their variable body size, which is called Peto’s paradox [178].

Many possible solutions to this paradox have been proposed. Indeed, increases in body mass have occurred many times during the evolution of life, and different mechanisms for suppressing tumors should have evolved independently, since an increased risk of cancer may exert a negative pressure on the continuation of the species. One solution is the greater effectiveness of tumor suppressor genes. Elephants have 20 copies (40 alleles) of *Tp53* genes [179], and naked mole rats have a supersensitive CDKN2A pathway [180,181]. The lower metabolic rate of large animals provides another solution to the paradox. High energy metabolism is associated with increased production of reactive oxygen species and other pro-tumorigenic chemicals, leading to a high mutation rate, whereas the metabolic rate generally scales with body mass to the 3/4 power; therefore, cells in larger animals tend to have a lower metabolic rate and less chance of tumorigenesis [182]. Other hypotheses include stronger immunocompetence against tumor cells and shorter telomeres that would limit the number of cell divisions in larger animals, although more evidence is needed to test these possibilities.

A recent study found a positive association between body size and cancer mortality after adjusting for gestational period length [176]. The study suggests that Peto’s paradox is not a paradox when considering the protective effect of the gestational period, the identity of which remains to be elucidated. The link between gestational time and cancer risk may be explained by slow cell proliferation, resulting in a low mutation rate in the fetus; maternal protective factors, such as hormones and the immune system; and the smaller tissue stem cell pool, which takes longer to generate a body. Another recent study also found that the body size and cancer risk do correlate, and that species that have experienced a more rapid evolutionary increase in body size have a low risk of cancer, even at the same body size today [183]. In the context of the mathematical models of carcinogenesis, the above hypotheses suggest that larger animals should have a lower number of tissue stem cells, a lower mutation rate, or a lower clonal expansion rate than expected.

### 5.3. Diversity in Radiation Cancer Risk Among Mammals

Numerous studies have been conducted on radiation carcinogenesis in humans and animals, and efforts have also been made to establish data archives, including experiments on mice, rats, dogs, rabbits, and monkeys [184,185,186]. Examples of studies using animals other than mice and rats include an experiment in which rabbits were exposed to 4.4–14.1 Gy and observed until natural death [187] and an experiment in which monkeys were exposed to 2.8–8.6 Gy of X rays or 2.3–4.4 Gy of neutrons and observed for their lifetime [188]. Radiation carcinogenesis experiments using hamsters [189], chickens [190], medaka fish [191], and *Drosophila* [192] have also been reported. Nevertheless, much of the nonhuman evidence still comes from experiments on mammals.

**Figure 6:**
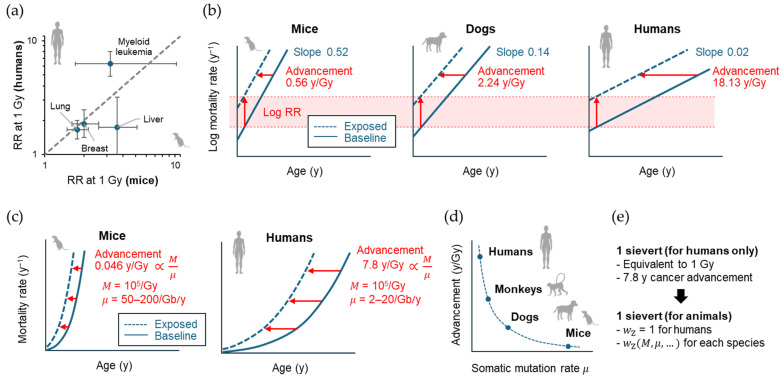
Historical development in interspecies comparison of radiation cancer risk. (**a**) Comparison of relative risks (RRs) between mice and humans by Storer et al. [171]. RRs for different cancer sites at 1 Gy are shown as points, confidence intervals (95% for mice, 90% for humans) are shown as error bars. The dashed line indicates the identity function (*y* = *x*). (**b**) Schematic of the comparative analysis by Carnes et al. [172], showing semi-log plots for the fitted exponential relationship between age in years (y) and cancer mortality rate. Vertical and horizontal arrows indicate radiation-related upward and leftward shifts corresponding to log RR and advancement of cancer mortality, respectively. (**c**) Schematic of the comparative analysis by Imaoka et al. [69] showing the fitted power function relationship between age and cancer mortality rate. Horizontal arrows indicate advancement of cancer mortality, which is theoretically proportional to the ratio of *M* (total radiogenic mutation) to *μ* (somatic mutation rate). (**d**) Hypothesis on the inverse proportionality between mammalian somatic mutation rate and advancement of mortality from radiation-related cancer. (**e**) Proposed species-universal effective dose. $w_{Z}$, zoological weighting factor.

Efforts have long been made to compare the results from different species (including humans) and to find underlying universal features. In 1989, Storer et al. [193] reported the possibility that the ERR/Gy for mortality from cancers of various organs coincide between mice and humans. The authors used data from 9763 female and male mice of four strains that received 0, 0.5, 1 or 2 Gy of γ rays at 10 weeks of age and calculated the “age-adjusted” mortality rate from various types of neoplasms. They found that ERR models were generally more acceptable than EAR models, and that the estimated ERR/Gy values for lung cancer, breast cancer, liver cancer, and leukemia were in good agreement with those reported for Japanese atomic bomb survivors [193] (Figure 6a). The follow-up period of the Japanese atomic bomb survivor cohort was short at that time, and therefore, the modification of risk by age at exposure and attained age was not fully discussed, although how to adjust for the different time scales between species is an important issue. The possibility that similar ERR values hold across species needs to be revisited using the recent atomic bomb survivor data.

In 2003, Carnes et al. [194] reported a reanalysis of the solid cancer mortality data from 3434 B6CF1 mice (exposed at ~110 days of age, 0.22–7.56 Gy) and 354 beagle dogs (exposed at 10–12 months of age, 0.75 or 2.25 Gy) and compared the results with those of atomic bomb survivors (limited to those exposed at 15–40 years of age to match the experiments, 0.01–4.59 Gy) using an empirical Gompertz model (i.e., risk proportional to the exponential of age). On a semi-logarithmic plot, the baseline cancer mortality rate had species-dependent slopes against age. The effect of radiation was interpreted as an upward and leftward shift in the graph, indicating the logarithm of RR and advanced occurrence of cancer death, respectively (Figure 6b). The upward shift per dose (i.e., log RR at 1 Gy) was consistent among the three species (0.0027 ± 0.0003 Gy^−1^), whereas the leftward shift was species-dependent (0.40 ± 0.12 for male mice, 0.72 ± 0.08 for female mice, 2.24 ± 0.44 for female dogs, and 18.13 ± 2.71 for humans; mean ± SE in years Gy^−1^; Figure 6b). This study confirmed the observation by Storer et al. [193] that the RR is a more universal measure of radiation cancer risk across species. On the other hand, the result can be interpreted in a somewhat different way—what is universal is the length of time by which cancer mortality advances, adjusted by the intrinsic cancer rate for each species. A limitation of this study is its use of an RR model that is independent of attained age and follows an exponential dose response across the entire dose ranges. However, a more recent study supports that the RR follows a linear or linear-quadratic dose response up to 2 Gy and decreases with attained age [195]. Thus, it remains to be seen whether the universality of the RR holds when an updated model is used.

In 2024, Imaoka et al. [89] reported their reanalysis of the cancer/leukemia mortality data from 2837 female B6C3F1 mice exposed to 0.02–8 Gy of γ rays acutely or chronically from 35 to 365 days of age and from 50,924 women exposed to 0.005–2 Gy in the atomic bomb survivor cohort [195]. Fitted to an empirical risk model often used in epidemiology, the dose response of the ERR showed a significant upward curvature in both species. When age at exposure and attained age were adjusted to ~35% and ~85% of the lifespan of each species (i.e., 84 years for humans and 2.36 years for mice), the dose response coefficients differed by ~10-fold between the species (humans, 0.22 *D* + 0.27 *D*^2^, exposed at 30 years, attained age 70 years; mice, 0.044 *D* + 0.015 *D*^2^, exposed at 0.8 years, attained age 2 years; where *D* is the dose in Gy). Thus, this study suggests that the idea of a universal ERR per unit dose across species may not hold in this case.

### 5.4. Mechanism of Diversity

The study by Imaoka and colleagues [89] also used a biologically based mathematical model of multistage carcinogenesis and found a clue to understanding the mechanism of species diversity in radiation cancer risk. Therein, the model predicted that the advancement of cancer mortality corresponds to a period of time equal to the time it would take for the spontaneous mutation process to generate the same amount of cancer driver mutations as those generated by exposure. That is, if we let *μ* be the spontaneous mutation rate and *M* be the total amount of radiogenic mutation, *M*/*μ* dictates the advancement of cancer mortality (Figure 6c). When fitted to the above mouse and human data, the length of advancement followed a linear dose response, in contrast to the ERR, with ~100-fold species difference (7.8 [95% CI, 5.3 to 10.3] and 0.046 [95% CI, 0.025 to 0.066] years Gy^−1^ for humans and mice exposed at ~35% of their lifespan, respectively). The modification of the length of advancement by age at exposure was consistent across species when standardized by their lifespan. Published data indicate that a mutation in the human *HPRT* and mouse *Hprt* genes occurs in ~1 in 10^5^ (in most cases 10^4^–10^6^) cells irradiated at 1 Gy, suggesting that *M* does not differ substantially between species. On the other hand, the recent development of single-cell deep sequencing technology has made it possible to measure the somatic mutation rate by analyzing the whole exome or genome of single somatic cells from organisms of different ages. Using this technique, studies have estimated somatic mutation rates in the range of 2–20 Gb^−1^ year^−1^ in humans and 50–200 Gb^−1^ year^−1^ in mice, supporting a more than 10-fold (possibly up to 100-fold) higher value for *μ* in mice. Thus, much of the ~100-fold higher value for the length of advancement per Gy (*M*/*μ*) in humans is explained by the common radiogenic mutation (*M*) and species-specific somatic mutation rates (*μ*). Thus, the study provides mechanistic support for the notion proposed by Carnes et al. [194] that the length of advancement of cancer mortality is universal among species when corrected for their intrinsic cancer rate, and predicts that the length of advancement of cancer mortality per Gy is inversely proportional to the somatic mutation rate *μ* (Figure 6d).

In the above analysis, the radiogenic mutation rate was derived from only one gene, whereas it is possible that the radiogenic mutation may vary between genes. It is thus warranted to explore a single-cell-based analysis for radiation-induced mutation rate across species. In fact, some results exist for mouse and human cells [72,196]. It would be feasible to extend the mathematical analysis of Imaoka and colleagues to other species including rats, dogs, and monkeys [184,185,186]. Although the above analysis only indicates the relevance of the species-specific somatic mutation rate, further analyses may indicate the relevance of other factors such as the stem cell pool size, clonal expansion rate, species-specific set of driver mutations, etc.

## 6. Perspectives

We have reviewed the variability in radiation cancer risk at different scales—cell/tissue type involved, physiological, environmental, genetic features of individuals, and animal species. A key finding herein is the common set of mechanisms driving the diversity across scales—summarized as diversity in stem cell pool size, mutation rate, clonal expansion rate, driver mutation repertoire, cellular epigenetics, radiation-induced tissue microenvironmental changes, and radiation-induced cell death.

One practical benefit of considering such diversity is that it provides mitigation measures by placing the diversity under control. Examples of such interventions include lifestyle changes that reduce radiation risk and the administration of protective chemicals that mimic lifestyle changes [197]. There is also the potential for new opportunities inspired by the trans-scale insights (e.g., deliberate control of epigenetics to mimic the cell types that have low radiation cancer risk). The trans-scale view will raise new questions about the species differences in radiation risk. For example, lower vertebrates (e.g., chickens [190] and medaka fish [191]) appear to have different distributions of organs at risk, raising the question of how this difference is determined. Another question is how the diversity in physiology, environment, and genetics of individual animal species relates to the interspecies diversity in radiation risk. Trans-scale insights also have implications for radiological protection. Diversity among tissues requires the use of the tissue weighting factor in radiological protection. Likewise, diversity among certain individual traits may necessitate novel weighting factors (e.g., for age and sex) in situations requiring special precautions for high exposure (e.g., medical procedures, emergency work, and space missions).

Notably, the diversity of radiation cancer risk among mammalian species should be considered in radiological protection in the veterinary field [173]. This issue is also relevant to environmental protection because the impact of cancer mortality on wildlife may be greater than previously expected [174,175]. Current radiological protection relies heavily on the concept of the effective dose, which is expressed in sieverts (Sv). This value is intended to express stochastic effects, much of which are cancer risks, on humans. A comprehensive understanding of the biological law governing species-specific radiation cancer risk has the potential to facilitate the development of a novel concept of the species-universal effective dose (Figure 6e). From this perspective, it is interesting to consider how some animals adapt to the high natural radiation levels present in their environment (e.g., 90–700 mGy/y to the lung in high radon environments) [198].

## 7. Conclusions

Taken together, trans-scale insights into the diversity of radiation cancer risk propose several common mechanisms underlying the diversities at different levels including cell/tissue, individual, and species scales. A balanced consideration of this diversity is the key to maintaining the reliability of radiological protection of humans, companion animals, and the environment.

## Figures and Tables

**Table 1:** Neutron and carbon-ion RBE in animal carcinogenesis of various tissues.

Tissue	Neutrons *^a^*			Carbon Ions		
	Energy (MeV)	Dose (Gy)	RBE	Beam type	Dose (Gy)	RBE
All solid	3.1	0.13–1	5–8	SOBP	0.43	1–12 [53]
	1 and 2.5	1.8–5.5	3–4			
Brain	2	0.024–0.49	7.1–21.5 *^b^* [54]	Plateau	0–0.5	4.1–4.3 *^b^* [55]
Breast	—	0.001	33	SOBP Plateau	0–2.0 0–2.0	2–10 [56] 0.2–2.8 *^b^* [57]
	0.5	0.001	15			
	2.43	0.001	100			
	2	0.049–0.97	7.5–26.3 *^b^* [58]			
Lung	—	0.001	19, 23–24	Plateau	0–4.0	1.1, 2.6 *^c^* [59]
	1.6–2.1	0.016–0.1	30–50			
	—	0.1–0.25	25–40			
	7.5	<1	4.5, 7.4 *^c^*			
	2	0–0.97	4.8, 4.6 *^c^* [59]			
Liver	0.4	0.09–0.17	13–28 *^b^*	—	—	—
	2.13	0–2.0	15, 2.5 *^c^*			
Small intestine	—	0.5–1.0	2–8 [60]	Plateau	0.1–2.0	1.4–3.7 [61]
Colon	—	—	—	Plateau	0.1–2.0	3.3–8 [61]
Kidney	—	—	—	Plateau	0.4	1.1 [62]
Soft tissue	—	—	—	SOBP	5–65 *^d^*	2.2 [63]
Bone marrow	1 and 5	0.001	1.8			
	—	0.001	2.8, 13			
	0.4	0–0.4	2.3			
	2	0–0.97	2.1 [64]			

*^a^* Information is taken from the International Agency for Research on Cancer [48] unless otherwise noted. *^b^* Depends on age at exposure. *^c^* Male and female, respectively. *^d^* 1–16 fractions. —, not reported. SOBP, spread-out Bragg peak. Shaded columns indicate information on neutrons.

## Data Availability

The original contributions presented in this study are included in the article/supplementary material. Further inquiries can be directed to the corresponding author(s).

## Supplementary Materials

The following supporting information can be downloaded at: https://www.mdpi.com/article/10.3390/biology14081025/s1, Supplementary material file: original data used.

## Abbreviations

The following abbreviations are used in this manuscript:

3D	Three-dimensional
CI	Confidence interval
DSB	Double-strand break
EAR	Excess absolute risk
ERR	Excess relative risk
LET	Linear energy transfer
RBE	Relative biological effectiveness
RR	Relative risk
SOBP	Spread-out Bragg peak
